# Molecular Characterization of Equine-like G3P[8] Rotavirus Strains Detected in South Korean Children

**DOI:** 10.3390/v17111488

**Published:** 2025-11-10

**Authors:** Yunhee Jo, Minji Lee, Deog-Yong Lee, Myung-Guk Han, Sun-Whan Park

**Affiliations:** Division of Viral Diseases, Department of Diagnosis and Analysis, Korea Disease Control and Prevention Agency, Cheongju 28159, Republic of Korea

**Keywords:** equine-like G3P[8] rotavirus, reassortant strain, DS-1-like genomic constellation, molecular epidemiology, vaccine effectiveness, Republic of Korea

## Abstract

The equine-like G3 rotavirus is a reassortant strain in which an animal-derived G3 genotype has recombined with a human-origin genetic backbone. Recently, this strain has spread across Asia and Europe. In this study, the *VP7* and *VP4* genes, along with the genomic backbone of 39 G3P[8] rotavirus strains detected in South Korean children with acute gastroenteritis between 2017 and May 2025, were analyzed. All strains harbored the equine-like G3 genotype for the *VP7* gene, and segmental genotyping of *VP7*, *VP4*, and *VP6* suggested that these strains possessed a DS-1-like genetic backbone (I2-R2-C2-M2-A2-N2-T2-E2-H2). All *VP4* genes were confirmed as P[8]. Phylogenetic analysis revealed that these strains clustered with previously reported equine-like G3 strains from various regions, including East Asia. Although individual vaccination records were not available in the EnterNet surveillance data, the overall detection rate of rotavirus infection has declined following vaccine introduction. Nevertheless, equine-like G3P[8] strains have continued to appear sporadically in Korean children, underscoring the importance of ongoing genomic surveillance in the post-vaccine era. Overall, these findings indicate that equine-like G3P[8] strains with a DS-1-like backbone have been circulating for several years in the pediatric population in South Korea, offering important insights into vaccine effectiveness and the surveillance of reassortant rotaviruses.

## 1. Introduction

Group A rotavirus (RVA), a member of the genus Rotavirus within the family Reoviridae, is a leading cause of acute gastroenteritis in children under 5 years of age worldwide, accounting for more than 200,000 deaths annually. Morbidity and mortality rates are particularly high in countries lacking national rotavirus vaccination programs [1,2]. RVA, belonging to the family *Reoviridae*, has a segmented genome consisting of 11 double-stranded RNA segments [3]. Among these, *VP7* (G genotype) and *VP4* (P genotype) are major outer capsid proteins that function as neutralization antigens and are critical for genotype classification [4,5]. Since the emergence of the equine-like G3P[8] strain in Germany in 2012, similar strains have been reported in Japan, China, Thailand, and Brazil, suggesting widespread inter-species reassortment and global dissemination.

In South Korea, sporadic detections of G3 strains have been reported in recent national surveillance, showing a similar circulation pattern to those observed in neighboring Asian countries. This contextual comparison underscores the global circulation and periodic re-emergence of equine-like G3P[8] strains.

The G3 genotype was widespread globally prior to the 1990s [6], but its prevalence declined following the introduction of rotavirus vaccines [7,8]. In South Korea, nationwide insurance-based surveillance showed a marked decline in rotavirus-associated hospitalizations after vaccine introduction [9] However, the EnterNet surveillance system does not record individual vaccination status, limiting direct assessment of vaccine effects while highlighting the need for continuous genotype monitoring. However, in recent years, a distinct lineage known as equine-like G3P[8] has emerged in several countries. This lineage, characterized by an equine-origin *VP7* gene and the DS-1-like genomic constellation (I2-R2-C2-M2-A2-N2-T2-E2-H2) [10,11], has been sporadically detected in South Korea according to EnterNet surveillance data (2014–2024). These findings suggest continuous circulation of reassortant rotavirus strains in the post-vaccine era. This genotype constellation likely originated through reassortment between animal- and human-derived gene segments.

Equine-like G3P[8] strains have been identified in several countries, including Indonesia, Vietnam, Japan, China, Venezuela, and Mozambique, indicating ongoing transboundary spread of this lineage [10,11,12]. Some of these strains exhibit genetic differences in antigenic regions relative to vaccine strains, raising concerns about potential vaccine escape [13].

In South Korea, G3P[8] strains were detected in a survey conducted from 2019 to 2023 [14]; however, molecular genetic studies on equine-like lineages remain scarce. To date, there have been no reports of the isolation or genomic characterization of equine-like G3P[8] strains in South Korea. Therefore, in this study, 39 G3P[8] rotavirus strains isolated from children under 5 years of age in South Korea between 2017 and May 2025, focusing on the *VP7* and *VP4* genes, were analyzed. Additionally, *VP6* gene analysis was performed on 10 strains with sufficient RNA quality collected within the past 5 years. This study aimed to investigate the temporal distribution and genetic characteristics of equine-like G3P[8] rotavirus strains detected in Korean children with acute gastroenteritis between 2017 and 2025 and to compare their genomic constellations with previously reported global strains.

## 2. Materials and Methods

### 2.1. Ethics Statement

As anonymized stool specimens collected for public health surveillance purposes were used in this study, ethical approval and individual informed consent were not required.

### 2.2. Sample Collection and Processing

In this study, stool samples of children under 5 years of age with acute gastroenteritis were collected from 76 medical institutions and 18 regional public health laboratories across South Korea between 2017 and May 2025 through the national EnterNet surveillance system. These specimens were initially screened for group A rotavirus at the regional public health laboratories.

Samples that tested positive for RVA were further genotyped using one-step RT-PCR assays targeting the *VP7* (G type) and *VP4* (P type) genes according to the national surveillance protocol.

Based on these genotyping results, specimens identified as G3P[8] were selected for the present study. The *VP7*, *VP4* and *VP6* genes of these samples were newly analyzed in this study to confirm their genetic characteristics.

Information on individual patient age and symptom severity was not available because anonymized stool specimens were collected through the national EnterNet surveillance system. Therefore, the study could not evaluate clinical severity based on the WHO grading criteria for acute gastroenteritis.

Approximately 1 g of each stool specimen was mixed with 9 mL of sterile phosphate-buffered saline, homogenized for 3 min, and centrifuged at 12,000 rpm for 10 min at 4 °C to obtain the supernatant.

### 2.3. RNA Extraction and RT-PCR

Viral RNA was extracted from 10% (stool/phosphate-buffered saline) suspensions using the NucloMag Viral RNA Isolation Kit (Macherey-Nagel, Düren, Germany) according to the manufacturer’s instructions. Reverse transcription and amplification of the *VP7*, *VP4*, and *VP6* genes were performed using the HyQ^TM^ One-step RT-PCR premix kit (Dye plus) (iMOD-001TD; SnC, Seoul, Republic of Korea).

Primer sets for *VP7* and *VP4* were selected according to the World Health Organization (WHO) Manual of Rotavirus Detection and Characterization Methods [15]. The *VP6* primer set was adopted from Lin et al. (2008), who established RT-PCR assays for the determination of rotavirus *VP6* genogroups I and II [16]. Each 25 µL reaction mixture contained 12.5 µL of RT-PCR premix, 1 µL (10 pmol; final ≈ 0.4 uM) of each primer, 10.5 µL of RNA template, and nuclease-free water to volume.

The expected amplicon sizes were approximately 881 bp (VP7), 876 bp (VP4), and 824 bp (VP6). Primer sets were adopted from the WHO Global Rotavirus Laboratory Network protocol.

The *VP7* gene was amplified using the forward primer VP7-F (5′-ATG TAT GGT ATT GAA TAT ACC AC-3′) and reverse primer VP7-R (5′-AAC TTG CCA CCA TTT TTT CC-3′). The thermocycling conditions for the *VP7* gene were as follows: reverse transcription at 42 °C for 30 min, initial denaturation at 95 °C for 15 min, followed by 35 cycles of denaturation at 94 °C for 30 s, annealing at 42 °C for 30 s, and extension at 72 °C for 7 min. For *VP4* gene amplification, the forward primer Con3 (5′-TGG CTT CGC TCA TTT ATA GAC A-3′) and reverse primer Con2 (5′-ATT TCG GAC CAT TTA TAA CC-3′) were used. The thermocycling conditions were as follows: reverse transcription at 42 °C for 40 min, initial denaturation at 95 °C for 15 min, followed by 35 cycles of denaturation at 94 °C for 1 min, and final extension at 72 °C for 7 min. *VP6* gene amplification was conducted using the forward primer 6BEG.303 (5′-AAY GTR TGT ATG GAT GAR ATG-3′) and reverse primer VP6-R (5′-GTC CAA TTC ATN CCT GGT GG-3′). The RT-PCR conditions were as follows: reverse transcription at 42 °C for 60 min, initial denaturation at 94 °C for 1 min, followed by 35 cycles of denaturation at 94 °C for 1 min, 52 °C for 40 s, 72 °C for 1 min, and final extension at 72 °C for 7 min.

PCR products were confirmed by agarose gel electrophoresis using an electrophoresis system, and the verified amplicons were subsequently sent to an external sequencing provider (Macrogen Inc., Seoul, Republic of Korea) for purification and Sanger sequencing.

The obtained sequences were assembled and edited using MEGA v11.

All sequences generated in this study are currently being prepared for submission to GenBank and will be made publicly available upon acceptance of the manuscript.

Owing to limitations in sample quality and available resources, *VP6* gene analysis was performed on 10 analyzable strains selected from the 39 specimens.

### 2.4. Phylogenetic Analysis

The amplified *VP7*, *VP4*, and *VP6* gene sequences were analyzed using MEGA version 11 software. Phylogenetic trees were constructed using the maximum likelihood method with the Tajima–Nei nucleotide substitution model in MEGA version 11, after sequence alignment with the ClustalW algorithm. No outgroup was used because the analysis included only G3P[8] strains. Statistical support was assessed using 1000 bootstrap replicates. Reference sequences representing previously reported equine-like G3P[8] strains and other G3 lineages were retrieved from the GenBank database. Selection was based on availability of complete gene sequences (*VP7*, *VP4*, and *VP6*), relevance to previously reported equine-like lineages, and geographical and temporal representation. Some reference strains reported between 2013 and 2016 were included because they were the earliest representatives of the equine-like G3P[8] lineage and have been widely used in previous phylogenetic studies.

## 3. Results

### 3.1. VP7 and VP4 Gene Analysis

**Figure 1:**
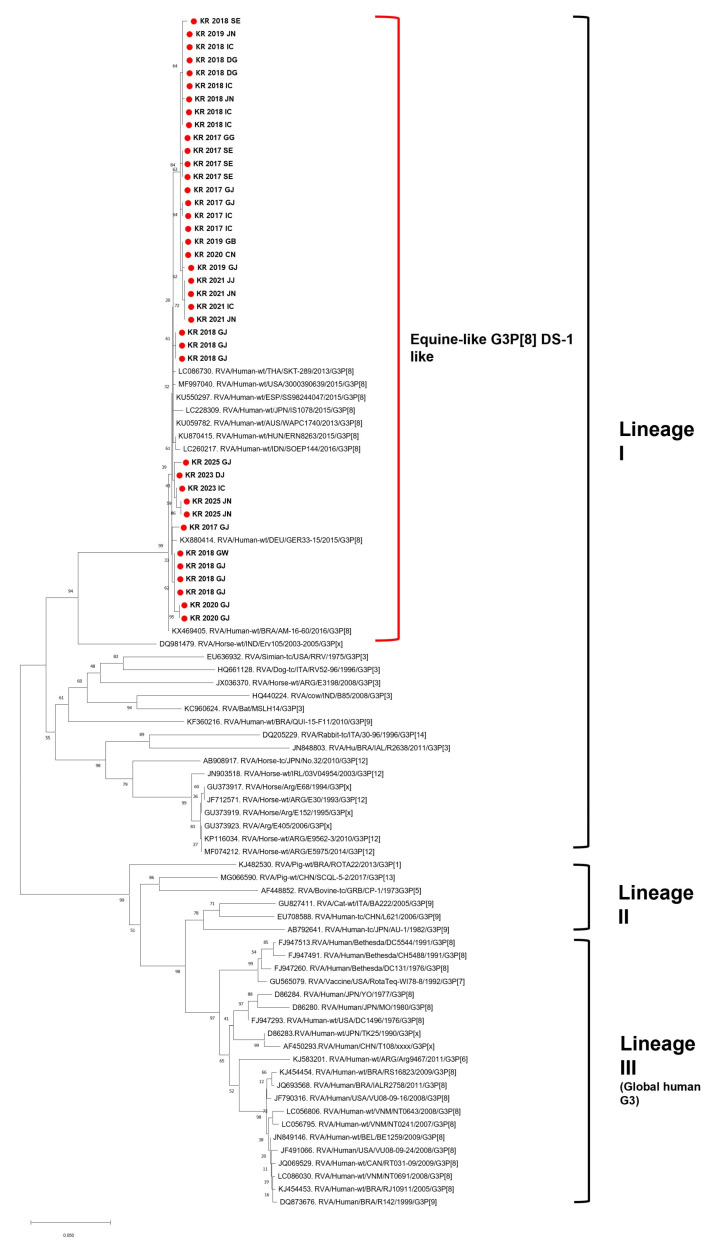
Phylogenetic analysis of the *VP7* gene of G3P[8] strains. Phylogenetic tree based on the *VP7* gene sequences of 39 G3P[8] rotavirus strains detected in South Korean children between 2017 and May 2025. All study strains clustered within the equine-like G3 lineage, exhibiting high nucleotide identity with previously reported strains from East and Southeast Asia. The tree was constructed using the maximum likelihood method with 1000 bootstrap replicates. Reference strains are labeled with GenBank accession numbers and country of origin. No outgroup was used. The scale bar indicates the number of nucleotide substitutions per site.

**Figure 2:**
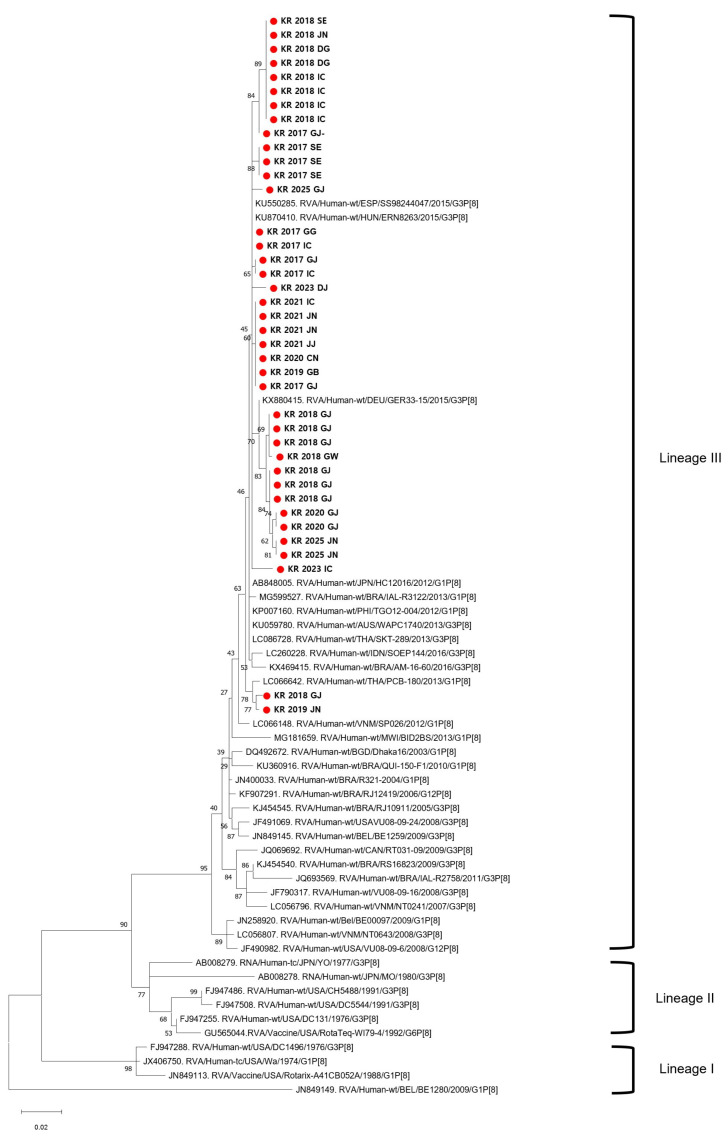
Phylogenetic analysis of the *VP4* gene of G3P[8] strains. Phylogenetic tree based on the *VP4* gene (P[8]) sequences of 39 rotavirus strains. All strains were classified as P[8]-Lineage III, clustering closely with equine-like G3P[8] strains previously reported in Japan and Indonesia. The analysis supports the reassortant nature of these strains, which combine an equine-derived *VP7* gene with a typical human-origin P[8] gene. The maximum likelihood method and 1000 bootstrap replications were used. Reference sequences are indicated with GenBank accession numbers. No outgroup was used because the analysis included only G3P[8] strains.

All 39 G3P[8] strains were confirmed to belong to the equine-like G3 lineage based on the *VP7* gene analysis, clustering within the same clade as international reference strains (Figure 1). The phylogenetic tree showed that the Korean strains grouped closely with equine-like G3P[8] strains recently reported in Asia, clearly separated from other G3 lineages. The *VP7* gene of Korean strains showed 99.0–99.6% nucleotide identity with reference strains from Thailand (LC086730) and Japan (LC260217), indicating high sequence homology within the East Asian cluster. In particular, they clustered with reference strains from Japan (2015-2016; LC086730, LC228309), Indonesia (2016; LC260217), Spain (2015; KU550297), Hungary (2016; KU870415), and Australia (2013; KU059782), indicating close genetic relatedness among East Asian equine-like G3P[8] strains. Because the analyzed samples were pre-identified as G3P[8], all VP4 sequences were, as expected, classified as P[8]-Lineage III. The *VP4* genes of the Korean equine-like G3P[8] strains showed 98.8–99.5% nucleotide identity (pairwise distance 0.00–0.02) with equine-like G3P[8] reference strains from Hungary (KU870410) and Germany (KX880415), confirming their close genetic relationship within the same lineage. The Korean G3P[8] strains were grouped with reference equine-like G3P[8] strains from Spain (2015; KU550285), Hungary (2016; KU870410), and Germany (2015; KX880415), which belong to the same P[8]-Lineage III cluster. Phylogenetic analysis showed that the Korean strains clustered with equine-like G3P[8] strains from Asia and other regions, indicating a close genetic relationship across different geographic areas (Figure 2). These findings are consistent with the global characteristics of the equine-like lineage. These results indicate that the Korean strains belong to the equine-like lineage and possess a DS-1-like genomic constellation.

### 3.2. VP6 Gene Analysis

**Figure 3:**
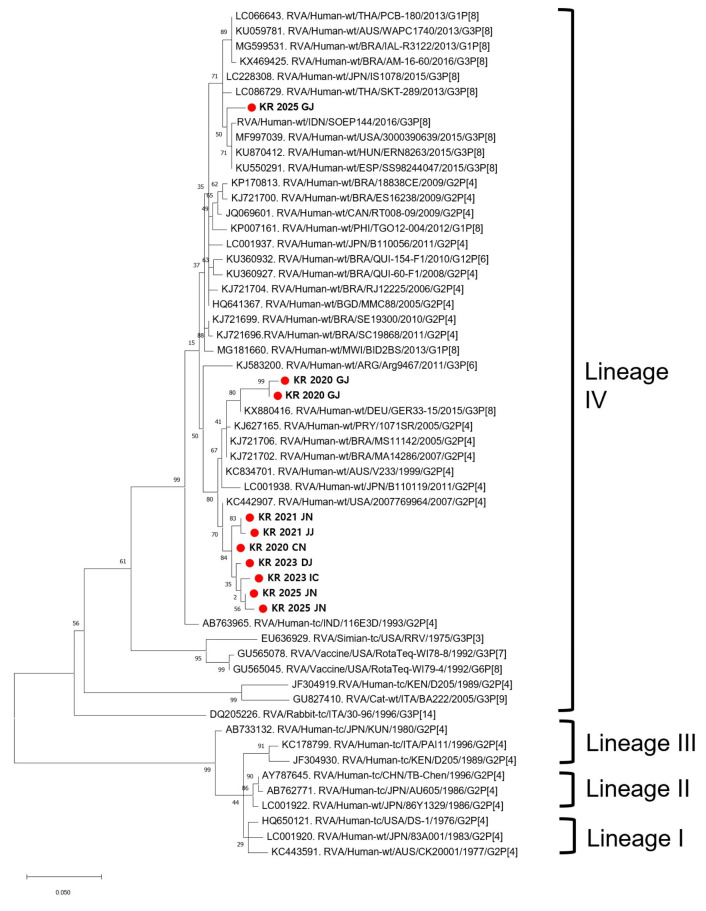
Phylogenetic analysis of the *VP6* gene of selected representative strains. Phylogenetic analysis of the *VP6* gene from 10 representative strains selected from the 39 G3P[8] samples. All strains were classified as genotype I2, consistent with the DS-1-like genome constellation. These results further support that the detected equine-like G3P[8] strains are reassortant viruses combining an equine-derived *VP7* gene with a DS-1-like human rotavirus backbone. The tree was constructed using the maximum likelihood method with 1000 bootstrap replicates. No outgroup was used because the analysis included only G3P[8] strains. The designation “Lineage I–IV” indicates four sub-clusters within genotype I2, which were defined in this study based on phylogenetic topology and do not represent an official lineage classification.

*VP6* gene analysis was conducted on 10 representative strains from the total of 39 strains owing to limitations in specimen quality and available resources. All 10 strains were classified as genotype I2 (Figure 3). They showed 98.0–99.0% nucleotide identity with reference I2-type strains from the United States (2014–2015; MF990939, KC442909) and Germany (2015; KX880416), forming a monophyletic cluster distinct from Wa-like I1-type strains. This finding is consistent with the *VP7* and *VP4* genotyping results and further supports their classification as equine-like strains with a DS-1-like genetic backbone.

## 4. Discussion

This is the first study to report the detection of equine-like G3 rotavirus strains in children under 5 years of age in South Korea between 2017 and 2025. Molecular characterization revealed that the identified G3P[8] strains belonged to the equine-like lineage and harbored the equine-like G3 *VP7*, P[8]-Lineage III *VP4*, and I2 *VP6* genotypes, constituting a DS-1-like genomic backbone. The equine-like G3 strains identified in this study exhibited high sequence homology both among the Korean strains and with reference equine-like G3P[8] strains from other countries in the *VP7* and *VP4* genes. Previous studies have reported that some equine-like G3P[8] strains possess genetic differences in antigenic regions compared to vaccine strains, raising concerns about immune escape and highlighting the need for continued immunological and neutralization-based evaluations [4,9]. The strains detected in this study exhibited high genetic similarity to equine-like G3P[8] strains previously reported in Australia, Asia, and Europe [10,11,14,15]. Additionally, the classification of *VP6* as genotype I2 supports the possibility that these strains have been circulating within local communities in South Korea for a sustained period, rather than representing a transient introduction.

The emergence of equine-like G3P[8] rotaviruses has been reported in several Asian and European countries, including Japan, China, Thailand, and Hungary, over the past decade. Consistent with those findings, the Korean equine-like G3P[8] strains in this study showed a DS-1-like genetic backbone (I2-R2-C2-M2-A2-N2-T2-E2-H2) combined with an equine-derived *VP7* gene. Recently, similar equine-like G3P[8] strains were detected in Brazil and Mozambique, showing comparable genetic constellations and evidence of inter-species reassortment between human and animal rotaviruses [17,18].

This study has certain limitations. The *VP6* gene analysis was restricted to 10 of the 39 strains owing to limited sample quality and resource constraints. Moreover, data on patient vaccination history and clinical symptoms were unavailable, preventing further analysis of potential associations. In addition, viral isolation and in vitro neutralization assays were not performed in this study, which limits the ability to directly assess the antigenic properties and vaccine escape potential of the detected strains. Nevertheless, the consistency between *VP6* and *VP7*/*VP4* genotyping results suggests that these 10 strains share key genetic features of equine-like lineage, although the analysis was limited to samples with available RNA. Future studies should incorporate full-genome sequencing and comparative analysis of antigenic epitopes to more precisely assess the vaccine escape potential, persistence, and transmission dynamics of equine-like strains in local populations.

## 5. Conclusions

This is the first study to report the detection of equine-like G3 rotavirus strains in stool samples of children with acute gastroenteritis in South Korea. This study confirmed that these strains harbor a DS-1-like genome constellation comprising equine-like G3 *VP7*, P[8]-Lineage III-*VP4*, and I2-*VP6* segments. These findings suggest a recent shift in the distribution of rotavirus genotypes in South Korea and provide valuable data for evaluating current vaccine effectiveness and monitoring emerging variant strains.

The detection of equine-like G3 strains also indicates the potential circulation of novel reassortant rotaviruses within the community, highlighting the importance of strengthening national rotavirus surveillance systems and developing early warning frameworks. Future immunological studies are warranted to assess cross-protection conferred by current vaccines and to evaluate the immune escape potential of these strains.

Future research should include nationwide, multi-center surveillance, neutralization assays at the cellular level, and full-genome sequencing to identify critical mutations. Such efforts would contribute to linking domestic data with international databases, enhancing global molecular epidemiological networks, and supporting the development of coordinated response strategies.

## Data Availability

The data presented in this study is available on request from the corresponding author.

## Abbreviations

The following abbreviations are used in this manuscript:

RT-PCR	Reverse transcription-polymerase chain reaction
RVA	*Rotavirus A*
